# Humans shape the year‐round distribution and habitat use of an opportunistic scavenger

**DOI:** 10.1002/ece3.6226

**Published:** 2020-04-15

**Authors:** Francisco Ramírez, Isabel Afán, Willem Bouten, Josep Lluís Carrasco, Manuela González Forero, Joan Navarro

**Affiliations:** ^1^ Departament de Biologia Evolutiva, Ecologia i Ciències Ambientals Facultat de Biologia Universitat de Barcelona Barcelona Spain; ^2^ Remote Sensing and GIS Laboratory (LAST‐EBD) Estación Biológica de Doñana CSIC Seville Spain; ^3^ Theoretical and Computational Ecology Institute for Biodiversity and Ecosystem Dynamics University of Amsterdam Amsterdam The Netherlands; ^4^ Departament de Fonaments Clínics, Bioestadística Facultat de Medicina Universitat de Barcelona Barcelona Spain; ^5^ Department of Conservation Biology Estación Biológica de Doñana (CSIC) Seville Spain; ^6^ Institut de Ciències del Mar - CSIC Barcelona Spain

**Keywords:** artificial night lights, compositional analyses, GPS tracking, scavenger, southwestern Spain, yellow‐legged gull

## Abstract

Research focused on evaluating how human food subsidies influence the foraging ecology of scavenger species is scarce but essential for elucidating their role in shaping behavioral patterns, population dynamics, and potential impacts on ecosystems. We evaluate the potential role of humans in shaping the year‐round distribution and habitat use of individuals from a typical scavenger species, the yellow‐legged gull (*Larus michahellis*), breeding at southwestern Spain. To do this, we combined long‐term, nearly continuous GPS‐tracking data with spatially explicit information on habitat types and distribution of human facilities, as proxied by satellite imagery of artificial night lights. Overall, individuals were mainly associated with freshwater habitats (mean proportion, 95% CI: 40.6%, 36.9%–44.4%) followed by the marine‐related systems (40.3, 37.7%–42.8%), human‐related habitats (13.5%, 13.2%–13.8%), and terrestrial systems (5.5%, 4.6%–6.5%). However, these relative contributions to the overall habitat usage largely changed throughout the annual cycle as a likely response to ecological/physiological constraints imposed by varying energy budgets and environmental constraints resulting from fluctuations in the availability of food resources. Moreover, the tight overlap between the year‐round spatial distribution of gulls and that of human facilities suggested that the different resources individuals relied on were likely of anthropogenic origin. We therefore provide evidence supporting the high dependence of this species on human‐related food resources throughout the annual cycle. Owing to the ability of individuals to disperse and reach transboundary areas of Spain, Portugal, or Morocco, international joint efforts aimed at restricting the availability of human food resources would be required to manage this overabundant species and the associated consequences for biodiversity conservation (e.g., competitive exclusion of co‐occurring species) and human interests (e.g., airports or disease transmission).

## Graphical abstract

We investigate the spatial distribution and habitat associations of a yellow‐legged gull population inhabiting a highly anthropogenic landscape, and throughout a complete annual cycle. The tight overlap between the spatial distribution of gulls and that of human facilities (as proxied by satellite imagery of artificial night lights) supports the high dependence of this species on human‐related resources. Gulls relied differently on different types of human food subsidies throughout the annual cycle as evidenced by observed changes in habitat use.

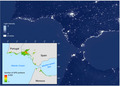

## 1 INTRODUCTION

Over millennia, humans have altered ecosystems through habitat destruction, pollution, species extinctions, and biological invasions, although these impacts have been exacerbated recently during the Anthropocene (McCauley et al., 2015; Rockström et al., 2009; Steffen et al., 2011). Besides these direct human impacts, food subsidies provided to wildlife and generated by human activities (e.g., refuse dumps, discards from fisheries, livestock middens, or crop leftovers) have played a role in shaping the structure and function of recent communities and ecosystems (Oro, Genovart, Tavecchia, Fowler, & Martínez‐Abraín, 2013). These abundant and predictable trophic resources have widely benefited scavenger species, because their opportunistic and flexible behavior has permitted efficient exploitation (Moleón et al., 2014; Oro et al., 2013). The efficient exploitation of anthropogenic food subsides has directly contributed to the expansive dynamics (i.e., increasing population sizes) of scavenger populations all over the globe (Newsome et al., 2015). Indirectly, these expansive dynamics have resulted in competitive exclusion processes with co‐occurring, less adaptable and often endangered species, leading to biotic homogenization worldwide (Olden, LeRoy Poff, Douglas, Douglas, & Fausch, 2004). Our understanding on the behavioral patterns of scavenger species, their population dynamics and their potential impacts on highly anthropogenic landscapes necessarily pass through a proper comprehension on their degree of dependence on human food subsides (Oro et al., 2013).

There are a number of scavenger species from a wide range of taxa that can benefit from anthropogenic food subsidies, both in marine and terrestrial ecosystems (see Oro et al., 2013 and references therein). Most studies on the foraging ecology for scavenger species have focussed on the spatial dimension and asses how spatially segregated populations adapt their feeding preferences, spatial distribution, or habitat usage to locally available food subsidies of anthropogenic origin during particular, often short time periods (e.g., reproduction; Baruch‐Mordo, Webb, Breck, & Wilson, 2013; Mendes et al., 2018; Ramos, Ramírez, Sanpera, Jover, & Ruiz, 2009a). However, the availability of these food resources can change both in space and time following spatiotemporal patterns in human activities, and opportunistic scavengers can potentially shape their foraging activity and searching processes to adapt to these varying circumstances (Gilbert et al., 2016; Newsome et al., 2015; Wong & Candolin, 2015).

Some gulls (*Larus *spp.) are widely considered as paradigmatic examples of species that benefit from anthropogenic food subsidies (Bécares et al., 2015; Duhem, Roche, Vidal, & Tatoni, 2008; Ramírez et al., 2015; Ramos et al., 2009a). This is particularly the case for yellow‐legged gulls (*Larus michahellis*) in the southern regions of the European continent, where populations have exponentially increased over the last decades and throughout their distribution range, likely as the result of their ability to efficiently exploit organic matter from refuse dumps or discards from fisheries (Duhem et al., 2008; Martínez‐Abraín & Jiménez, 2016; Ramos, Ramírez, Sanpera, Jover, & Ruiz, 2009b). While its opportunistic behavior and adaptable nature is widely accepted, few studies have previously investigated fine‐scale seasonal changes in the foraging ecology of this species (Anderson et al., 2019; Ramos, Ramírez, Carrasco, & Jover, 2011; Van Donk, Shamoun‐Baranes, Bouten, Meer, & Camphuysen, 2019); thus constraining our comprehension on their degree of dependence on human food subsides.

Here, we used nearly continuous GPS‐tracking data to investigate the year‐round distribution and habitat use of individuals from a yellow‐legged gull population that inhabit a highly anthropogenic landscape (southeastern Spain). In particular, we evaluated if gull distributions were associated with human facilities and their specific resources (as proxied by satellite imagery of artificial night lights). Temporal changes in foraging habitat selection were analyzed via compositional analyses to account for partitioning among habitat categories. We hypothesized that both the spatial distribution and foraging ecology of this species would be shaped by multiple constraints imposed by varying energy budgets throughout the annual cycle and spatiotemporal patterns in the availability of anthropogenic food subsidies. We evaluated how human activities in a widely transformed and heterogeneous landscape can shape the foraging ecology of a typical scavenger species in both space and time. Besides their ecological interest within the current context of human impacts on ecosystems' structure and functioning (Oro et al., 2013), these results may also have important management implications, such as providing information about those habitats, and hence food subsidies, that may contribute the most to the expansive dynamics of this species (Martínez‐Abraín & Jiménez, 2016; Payo‐Payo et al., 2015; Vidal, Medail, & Tatoni, 1998).

## 2 MATERIALS AND METHODS

### 2.1 Model species and study area

The yellow‐legged gull is an overabundant species inhabiting the Mediterranean region (Vidal et al., 1998). This region includes classic examples of highly anthropogenic and human impacted marine and terrestrial systems (Ales, Martin, Ortega, & Ales, 1992; Green et al., 2017; Ramírez, Coll, Navarro, Bustamante, & Green, 2018). With increasing pressure of human population growth, yellow‐legged gulls increasingly scavenge for food sources of anthropogenic origin (Martínez‐Abraín & Jiménez, 2016; Mendes et al., 2018; Payo‐Payo et al., 2015). This has likely increased its population growth rate (Oro et al., 2013) and resulted in an exponential rise in population (Vidal et al., 1998). Several conservation and management problems have emerged as a consequence of these expansive dynamics, including negative impacts on human interests (e.g., airports, agriculture, fisheries, or disease transmission) and interacting species (e.g., through competitive inclusion or predation; Oro et al., 2013; Ramos et al., 2009b; Vidal et al., 1998). Consequently, considerable efforts have been made to investigate the foraging ecology of this species to identify and regulate the availability of anthropogenic food subsidies on which they rely (Mendes et al., 2018; Oro et al., 2013; Ramos et al., 2009a, 2009b). Ours is the first study to provide nearly continuous data on the distribution and habitat use of yellow‐legged gulls throughout a complete annual cycle.

**FIGURE 1:**
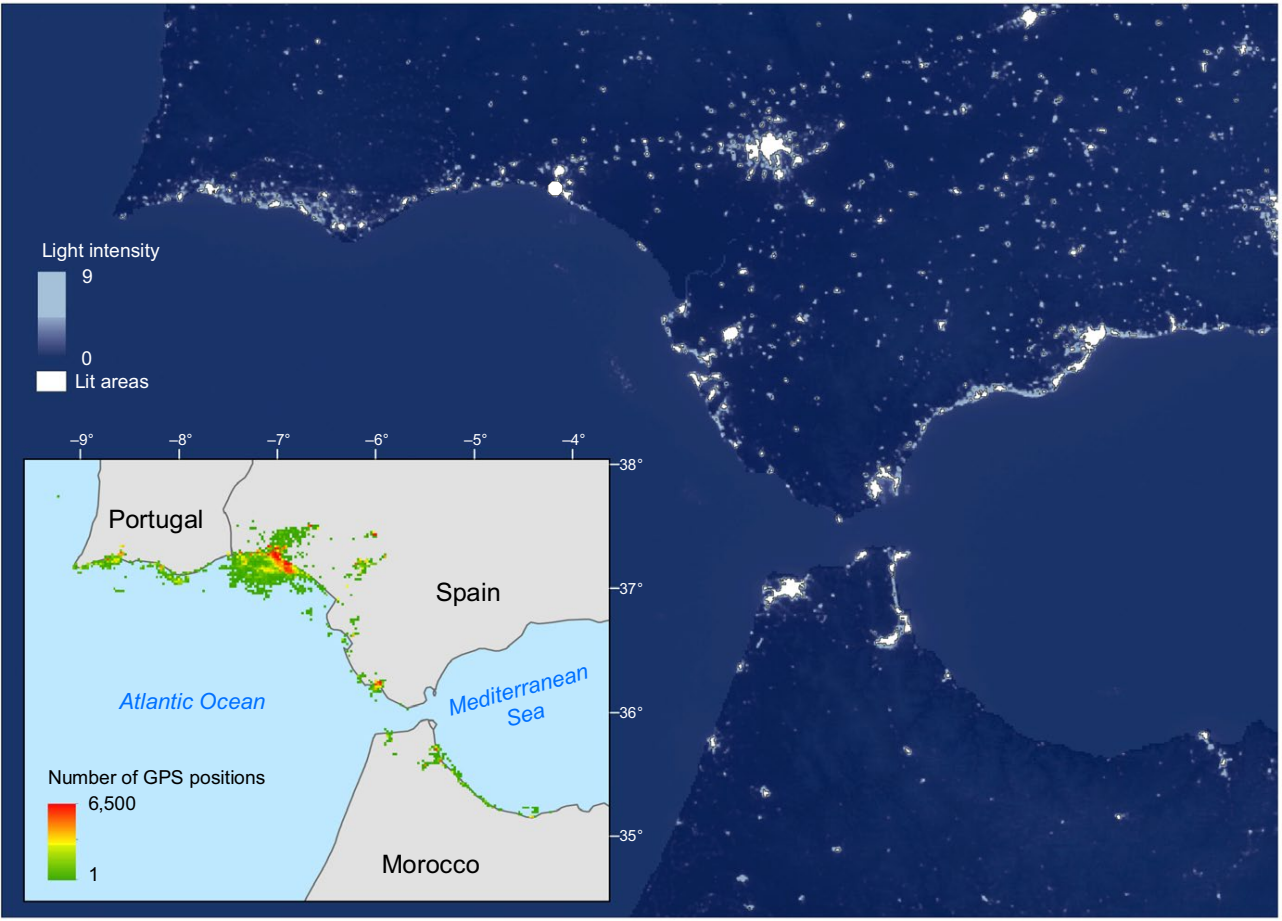
Study area and year‐round spatial distribution of yellow‐legged gulls equipped with GPS loggers during the 2015–2016 annual cycle. Color scale denotes the total number of GPS positions per pixel within a 2 × 2 km grid map out of the breeding period when individuals are no longer constrained by their central place foraging behavior. Gulls preferentially distributed themselves near human facilities and over a wide region that included coastal areas of Portugal, Spain, and Morocco. As a proxy for the spatial distribution of human facilities we used imagery products on human‐related lights at night from the Visible Infrared Imaging Radiometer Suite (see Section 2). Those pixels encompassing night light intensity (radiance) > 25 percentile (lit areas) are represented in white

We focussed on a yellow‐legged gull colony (about 300 breeding pairs) breeding in the protected Biosphere Reserve of Marismas del Odiel (Gulf of Cadiz, southwestern Spain), but dispersing over a wide region that includes coastal areas of Portugal and Morocco outside of the breeding period (Figure 1). This area has been largely impacted and modified for decades due to the development of human activities such as agriculture, fisheries, and coastal tourism (Green, Bustamante, Janss, & Fernández‐Zamudio, 2016; Ramírez et al., 2015; Serrano, 2006; Silva, Gil, & Sobrino, 2002). Thus, gulls inhabiting this area are provided with a wide range of food subsidies of anthropogenic origin that can potentially shape their foraging ecology and affect population dynamics (Table 1).

**TABLE 1:** Habitat categories used by our yellow‐legged gull population throughout the 2015–2016 annual cycle and potentially related food subsidies of anthropogenic origin (modified from Navarro et al., 2017; Oro et al., 2013)

Habitat domains	Habitat categories	Related food subsidies
Water domain	Freshwater habitats (wetlands, water ponds, fish farms, and salt pans)	Food production (e.g., fish)
		Resource facilitation (e.g., aquatic preys)
	Marine‐related systems (sea, estuaries, and beaches)	Fishery discards
Terrestrial domain	Human‐related habitats (dumps, fishing ports, and urban areas)	Organic refuses
		Bird feeders
	Terrestrial systems (agriculture areas and natural landscape)	Crop residuals
		Livestock carcasses Scavenging livestock food

### 2.2 Fieldwork procedure

During the incubation period of 2015 (April the 21st to May the 7th), 30 adult gulls were equipped with solar‐powered UvA‐BiTS GPS trackers (http://www.UvA‐BiTS.nl; Bouten, Baaij, Shamoun‐Baranes, & Camphuysen, 2013). GPS loggers were programmed on a 5‐min basis to acquire geographical locations, time (UTC), ground speed (km/h), and triaxial acceleration [surge (*x*), sway (*y*), and heave (*z*), measured at 20 Hz for 1–3 s, directly following a GPS fix] continuously throughout an entire annual cycle (i.e., GPS locations and acceleration data every 5 min from April 2015 to March 2016). Gulls were caught at nests using a walk‐in wire mesh trap, and equipped using wing harnesses constructed of tubular Teflon™ ribbon (Bally Ribbon Mills 8475‐0.25″). With a total mass of ca. 20 g, these devices represented <2% of birds' body mass [1,062 ± 20 g (mean ± *SD*)], thus preventing harmful effects on bird performance (Passos, Navarro, Giudici, & González‐Solís, 2010; Phillips, Xavier, & Croxall, 2003). Recorded data were transmitted remotely from devices to the UvA‐BiTS database when the birds were inside the reception area of a ground station located at the breeding colony (Bouten et al., 2013). The number of tracked birds, and hence recorded data, declined throughout the annual cycle likely due to loss of devices (two individuals removed the logger by cutting the harness), gull deaths (at least one gull was depredated by a red fox *Vulpes vulpes*), or technical constraints during data retrieval (i.e., insufficient connection time with the ground station to download all logged data or journeys with no return over the ground station). Ultimately, logger information was only available for six tracked birds at the end of the annual cycle (see Appendix S1 for our analyses on missing patterns).

### 2.3 Data analyses

To provide an overview of the distribution of gulls year‐round, we estimated the total number of GPS positions per pixel within a 2 × 2 km grid map covering a 50 km buffer area along the southern coast of the Iberian Peninsula and the northern coast of Morocco (21,974 grids). We excluded travelling locations (ground speed > 5 km/h; Navarro et al., 2016, 2017) and data for the breeding period (April–July 2015, based on the long‐term monitoring of this colony by the staff of the Biosphere Reserve of “Marismas de Odiel”), when individuals are largely constrained by their central place foraging behavior, to evaluate if gulls' distribution responded to human facilities (e.g., human settlements) and associated resources.

As a unique and continuous proxy to the spatial distribution of human facilities throughout the entire distribution range, we used satellite imagery of artificial night lights. Imagery products on human‐related lights at night from the Visible Infrared Imaging Radiometer Suite (VIIRS) were sourced online at the US National Oceanic and Atmospheric Administration (Earth Observation Group, NOAA National Centers for Environmental Information ‐NCEI‐: https://ngdc.noaa.gov/eog/viirs/download_dnb_composites.html, accessed on February 2019) as monthly composites for the August 2015–March 2016 period (i.e., the nonbreeding season). These products contain continuous information on artificial night light intensity (radiance in nanoWatts/cm^2^). Monthly products were aggregated to obtain a single snapshot on the distribution of night lights throughout the 2015–2016 nonbreeding season. Continuous data on light intensity were recategorized to produce a binary product informing on the spatial distribution of night light spots. Night light spots included those pixels encompassing night light intensity (radiance) >25 percentile (Figure 1). This threshold was selected through a visual inspection on the spatial overlap between estimated lit areas and available land cover information for Spain and Portugal (see below).

The estimated number of nontravelling GPS positions per 2 × 2 km pixel (including 1,000 randomly selected grids with GPS positions = 0) were fitted through a generalized additive model (GAM) with a negative binomial distribution and a log link function. As predictors, the model included the Euclidean distances from the centroid of selected grid cells to the nearest night light spot. Because gulls and human facilities preferentially occur near the coast (see Figure 1), the Euclidean distances of these centroids to the coastline were also included in the model to account for potential confounding factors. The model also included smoothed terms for the centroids' geographic coordinates to account for the spatial auto‐correlation.

To investigate habitat use by tracked gulls, we first excluded all those GPS positions encompassing passive behaviors based on triaxial acceleration data (see Shamoun‐Baranes, Bouten, Loon, Meijer, & Camphuysen, 2016) for nontravelling locations outside the breeding colony (out of a 500 m spatial buffer around tracked gulls' nests). Thus, we assumed that observed trends informed on temporal changes in habitat usage in terms of foraging, that is, searching for food and feeding. We identified the habitat used by overlapping the filtered locations with high‐resolution land cover information from Spain (SIOSE, Soil Information System of Spain, scale 1:10,000, Junta de Andalucía, last update 2013) and Portugal (COS, Carta de Uso e Ocupação do Solo, Direção‐Geral do Território, scale 1:25,000, last update 2015). These two products are based on aerial orthophotography and produced within the National Plans of Aerial Orthophotography following the EU INSPIRE directive (https://inspire.ec.europa.eu/). We constructed four habitat categories that summarized the main habitat selection of gulls: freshwater habitats, marine‐related systems, human‐related habitats, and terrestrial systems (see Table 1). Appropriate land cover information for Morocco was not available, so one habitat of these four categories was assigned to each filtered GPS‐position based on visual inspection of the most recent satellite images and orthophotography offered by Google Earth Pro 7.1.4.1529.

Based on recorded positions, we estimated the proportion of time spent at each particular habitat category per week (our unit of time). Resulting proportions were “ILR” (Isometric Log ratio Transformation) transformed following Egozcue, Pawlowsky‐Glahn, Mateu‐Figueras, and Barcelo‐Vidal (2003). Trends in habitat use (i.e., weekly changes in relative habitat proportions) throughout the annual cycle were modelled through Generalised Additive Mixed Models (GAMMs, Wood, 2006) with a negative normal distribution and an identity link function in three successive steps to account for the relative associations among habitat types. GAMMs included the individual as random factor and a two‐level fixed factor for evaluating differences between the breeding (April–July 2015) and the nonbreeding season (August 2015 – March 2016). The temporal trend was adjusted by applying P‐splines smoothing (Eilers & Marx, 1996). The significances of the P‐splines and the fixed effects were evaluated through *F*‐tests (Wood, 2006) and Likelihood Ratio Test (LRT), respectively. Habitat associations were evaluated through a hierarchical clustering (Ward's minimum variance method; Murtagh & Legendre, 2014) where similarities among habitats types were estimated using Centered Log‐Ratios (clr) of habitat proportions (Aitchison, 1986, Faith, 2015; see Appendix S2). We first evaluated the relative trends for the water (freshwater habitats and marine‐related systems) versus the terrestrial (human‐related habitats and terrestrial systems) domains. Subsequently, we evaluated relative trends within each domain; that is, freshwater habitats versus marine‐related systems and human‐related habitats versus terrestrial systems. Our approach assumes that missing information due to the decline in the number of tracked individuals throughout the study period had no associations with the response variables we were fitting (i.e., habitat usage); that is, that observed trends in habitat associations were not biased by the better performance of individuals (and/or tracking devices) preferentially occurring at particular habitats. We have no evidence that makes us suspect that such relationships may exist, and our analyses on missing patterns (Appendix S1) suggested that missing data for the entire annual cycle was random and, hence, that our approach to evaluate trends in habitat use based on maximum likelihood was suitable (Hedeker & Gibbons, 2006). Accordingly, observed trends in habitat usage were likely consistent among individuals. In any case, estimated habitat associations at the end of the study period must be taken with caution as they were driven by a reduced number of tracked individuals (Figure 3). Data analyses were conducted in R (R Core Team, 2017).

## 3 RESULTS

**FIGURE 2:**
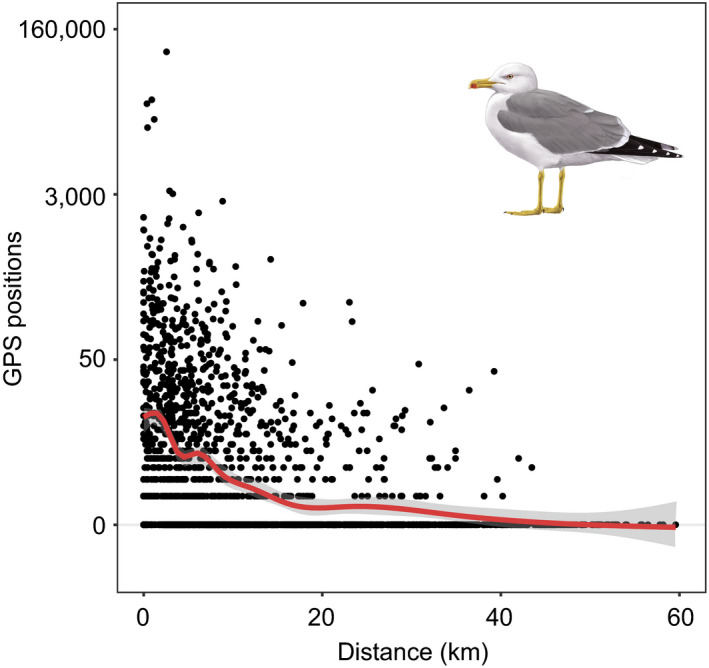
Observed relationship between the total number of nonbreeding (August 2015–March 2016) GPS positions per pixel within a 2 × 2 km grid map (logarithmic scale) and the Euclidean distances from the centroid of these grids to the nearest night light spot (night light intensity > 25 percentile). The draw of yellow‐legged gull was made by Martí Franch

**FIGURE 3:**
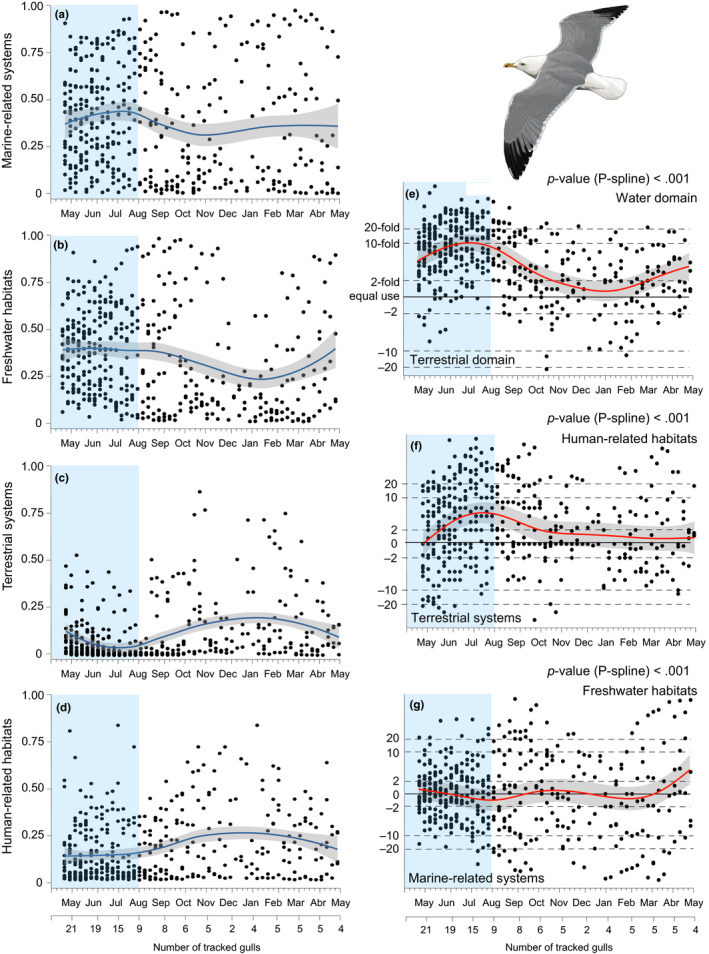
Habitat use by tracked yellow‐legged gulls throughout a complete annual cycle (blue vertical bars indicate the breeding period). (a–d) Temporal trends in the relative contributions of main habitat categories (see Table 1) to the overall habitat usage by gulls; that is, proportion of time associated to each habitat per week (scaled to 0–1 values). (e–g) Results of the compositional analyses showing habitat partitioning and associations among habitat categories. Trends in habitat partitioning were described in three successive steps to account for the relative associations among habitat types (see Section 2 and Appendix S2). The draw of yellow‐legged gull was made by Martí Franch

Up to 245,000 GPS locations occurred outside of the breeding season (see Figure 3 for the number of tracked individuals throughout the annual), when gulls' distribution was no longer constrained by their central place foraging behavior and individuals dispersed and reached transboundary areas at the southern Atlantic coast of Spain and Portugal, and the northern Mediterranean coast of Morocco (including 849 2 × 2 km grid cells, Figure 1). Our model to fit gull abundances during the nonbreeding period (see above) explained ca. 82% of observed deviance and showed low overdispersion (θ = 1.18), thus pointing to a good performance and to a consistency in observed associations throughout the nonbreeding period. In addition, the comparison between the full and the reduced model (including the Euclidean distance to coastline and the smoothed terms for the centroids' geographic coordinates) indicated a significant, additional effect of estimated distances to night lit areas (*χ* ^2^ = 55.8, *df *= 1, *p* < .001). Accordingly, gull distribution outside of the breeding period apparently followed the distribution of human settlements and human‐related facilities throughout the coastline (Figure 1), with the abundance of GPS locations increasing at a rate of 21.5%/km (95% CI: 15.4%–27.9%) as they approach to night lit areas (Figure 2).

We identified ca. 280,000 recorded positions with active behavior (based on accelerometry data) and incorporated habitat type, thus allowing our analyses on habitat usage throughout the annual cycle. Overall, tracked gulls were mainly associated with freshwater habitats (mean proportion, 95% CI: 40.6%, 36.9%–44.4%) followed by the marine‐related systems (40.3, 37.7%–42.8%), human‐related habitats (13.5%, 13.2%–13.8%), and terrestrial systems (5.5%, 4.6%–6.5%). However, these relative contributions to the overall habitat usage by gulls largely changed throughout the annual cycle (Figure 3). For instance, the relative contribution of habitats within the water domain (freshwater and marine‐related habitats) shifted from being ca. 10 times greater during the breeding season (from May to July) to roughly equal that for habitats within the terrestrial domain (human‐related habitats and terrestrial systems) beyond the breeding period (LRT *p*‐value < .001, Figure 3e). A similar pattern was found when comparing the relative contribution of human‐related habitats versus terrestrial systems, with the former being preferentially used during the breeding season (LRT *p*‐value = .01, Figure 3f). In contrast, gulls equally shared the use of freshwater habitats and marine‐related systems throughout the entire annual cycle (LRT *p*‐value = .88, Figure 3g). The individual random factor accounted for 45% (95% CI: 27%–59%), 48% (30%–63%), and 61% (44%–74%) of the total variance for the three latter relative trends, respectively.

## 4 DISCUSSION

We provide the first quantitative assessments of the spatial distribution and habitat associations of yellow‐legged gulls inhabiting a highly anthropogenic landscape, throughout a complete annual cycle and on a nearly continuous basis. Our results confirmed the opportunistic nature and flexible behavior of this species (Navarro et al., 2017; Payo‐Payo et al., 2015; Ramos et al., 2011; Ramos et al., 2009a), as suggested by the varying contributions of different habitat types to their overall habitat usage. Our results also supported the high dependence of this species on human‐related resources (Ramos et al., 2009a, 2009b), as demonstrated by the tight overlap between the spatial distribution of tracked gulls and that of human facilities. Combined, these results supported our expectations and suggest that individuals may adapt their foraging ecology to exploit different types of human‐provided food subsidies in response to (a) ecological/physiological constraints imposed by varying energy budgets throughout the annual cycle (Alonso, Almeida, Granadeiro, & Catry, 2015; Mendes et al., 2018), and (b) environmental constraints resulting from fluctuations in the availability of human‐related resources (Ramos et al., 2011).

Through industrial fisheries, fish farms, livestock, crops, and garbage dumps, humans provide animals with abundant and predictable food subsidies that have profoundly affected their dynamics and ecological traits (Oro et al., 2013). The annual distribution of our tracked gulls included a highly anthropogenic and heterogeneous landscape. In particular, this region is characterized by intense fishing and agricultural activities (Green et al., 2016; Serrano, 2006; Silva et al., 2002) and densely populated urban areas concentrated near the coast (Halpern et al., 2008; Ramírez et al., 2015). The tight overlap between the distribution of gulls and human facilities supports the reliance of individuals on subsidies of anthropogenic origin. Human settlements and different human activities may provide gulls with a wide range of food subsidies that they can efficiently exploit (Table 1 and Ramos et al., 2011, Navarro et al., 2017, Enners, Schwemmer, Corman, Voigt, & Garthe, 2018, Mendes et al., 2018). We suggest that the relative contributions of these diverse human food subsidies to the gulls' diet apparently changed throughout the annual cycle as inferred by observed trends in habitat usage. Complementarily, the increased light levels near human settlements may facilitate prey detectability and attract gulls to adjoining areas.

Gull diets are flexible and can largely vary depending on locally available resources (Enners et al., 2018; Mendes et al., 2018; Ramos et al., 2009a, 2011). The plasticity in their distribution and habitat selection was observed both at the population level and throughout the annual cycle. Indeed, we observed a large variability in the relative use of habitats among tracked individuals (explaining 45% to 61% of total variance), thus highlighting the population‐level plasticity typical of gull species (Corman, Mendel, Voigt, & Garthe, 2016; Enners et al., 2018; Mendes et al., 2018; Shaffer et al., 2017) and, potentially, the presence of individual foraging strategies within this population (Navarro et al., 2017). Despite this variability, the overall temporal trend in habitat use for this population suggested a clear shift toward an increasing use of food resources within the water domain (i.e., marine‐related systems and freshwater habitats) during the breeding period, when energy budgets of seabirds typically peak (Durant et al., 2005; Ramírez et al., 2010; Shaffer, Costa, & Weimerskirch, 2003). Reproduction may therefore impose an ecological/physiological constraint to gull foraging strategies as terrestrial resources may be deficient in the necessary nutrients for chick development (Alonso et al., 2015; Annett & Pierotti, 1999; Mendes et al., 2018).

A nonexclusive, alternative explanation to observed trends in habitat use may involve foraging adaptations to seasonal variations in habitat availability and resource abundances (Ramos et al., 2011). However, gulls preferentially occurred at permanent water masses such as tidal areas or artificial water ponds, fish farms, and salt pans (see https://global‐surface‐water.appspot.com/), whereas marine productivity in this area typically peaks during the winter (García Lafuente & Ruiz, 2007; Ramírez et al., 2015). Furthermore, human activities have profoundly altered natural ecosystem functioning and provide abundant, permanent and therefore predictable (both in space and time) food resources to gulls (Oro et al., 2013). Tourism is probably the only socio‐economic activity in our study area that peaks in the summer and decreases thereafter, and this may affect the feeding ecology of yellow‐legged gulls through the increasing consumption of human‐related resources (Ramos et al., 2009a, 2011). This scenario seems to be the case for our tracked yellow‐legged gulls, as the relative contribution of human‐related habitats (and therefore resources) was ca. ten times greater than that for terrestrial systems during the summer period. While adapting to varying energy budgets throughout the annual cycle, gulls may also fine‐tune their foraging ecology to temporal variations in the availability of human food resources.

By combining spatially explicit and nearly continuous information on the distribution and habitat use by these gulls, we have provided a more holistic view on the foraging ecology of this scavenger species, and on its likely dependence on anthropogenic food subsidies. We demonstrated that gulls were able to use a variety of habitats, and hence resources, as a likely response to both ecological/physiological (energy budgets) and environmental constraints (food availability). Moreover, the linkage between gull distributions and that of human activities suggests that these resources are likely of anthropogenic origin. Thus, food subsidies provided by humans throughout an entire annual cycle apparently play a key role in supporting this population. We therefore provide evidence supporting the idea that gull populations and their expansive dynamics may be controlled by regulating the availability of these food subsidies (Oro & Martínez‐Abraín, 2007; Payo‐Payo et al., 2015; Ramos et al., 2009b). However, the necessity of regulating human food subsidies can make a difference between highly regulated and developing countries (e.g., European vs*.* African countries), where high human densities are often coupled with less strict environmental policies. Owing to the ability of individuals to disperse and reach transboundary areas, international joint efforts aimed at restricting the availability of human food resources would be required to tackle this conservation issue beyond the interests and borders of sovereign states.

Anthropogenic food subsidies have altered the dynamics of this and other scavenger species for decades, with cascading effects across communities and trophic levels (Oro et al., 2013). Now that humans have started to restrict the availability of a range of these food subsidies (e.g., European Union Landfill Directive and Common Fisheries Policy), we must wonder how communities (as a whole) will respond when these resources are no longer available (Oro et al., 2013; Pons, 1992). Continuous monitoring of overabundant scavenger species through high‐resolution tracking data that allows comparison with specific local food subsidies would be desirable in order to identify, and potentially prevent, unwanted impacts on natural communities and human interests (Oro et al., 2013; Ramos et al., 2009a).

## CONFLICT OF INTERESTS

The authors declare no competing interests.

## AUTHOR CONTRIBUTION

**Francisco Ramírez:** Conceptualization (lead); Data curation (lead); Formal analysis (equal); Investigation (equal); Methodology (equal); Resources (equal); Validation (equal); Visualization (equal); Writing‐original draft (equal). **Isabel Afan:** Conceptualization (equal); Data curation (equal); Formal analysis (equal); Investigation (equal); Methodology (equal); Resources (equal); Writing‐review & editing (equal). **Willem Bouten:** Conceptualization (equal); Data curation (equal); Formal analysis (equal); Software (equal); Writing‐review & editing (equal). **Josep Lluís Carrasco:** Data curation (equal); Formal analysis (lead); Investigation (equal); Methodology (equal); Writing‐review & editing (equal). **Manuela González Forero:** Conceptualization (equal); Data curation (equal); Funding acquisition (lead); Investigation (equal); Project administration (equal); Writing‐review & editing (equal). **Joan Navarro:** Conceptualization (equal); Data curation (equal); Formal analysis (equal); Funding acquisition (equal); Investigation (equal); Methodology (equal); Project administration (equal); Writing‐review & editing (equal). 

## Supporting information

Fig S1

Fig S2

Fig S3

Appendix S1‐S2

## Data Availability

All data are available in a central PostgreSQL database at UvA‐BiTS (http://www.uva‐bits.nl/virtual‐lab).
