# Supplementary figures and images for: Humans shape the year‐round distribution and habitat use of an opportunistic scavenger
Source: Ecol Evol. 2020 Apr 15;10(11):4716–25. doi: 10.1002/ece3.6226 (PMC7297764; doi:10.1002/ece3.6226)

Number of sampled weeks

20  
15  
10  
5  
0

May Jun Jul Aug Sep Oct Nov Dec Jan Feb Mar Abr May

Week

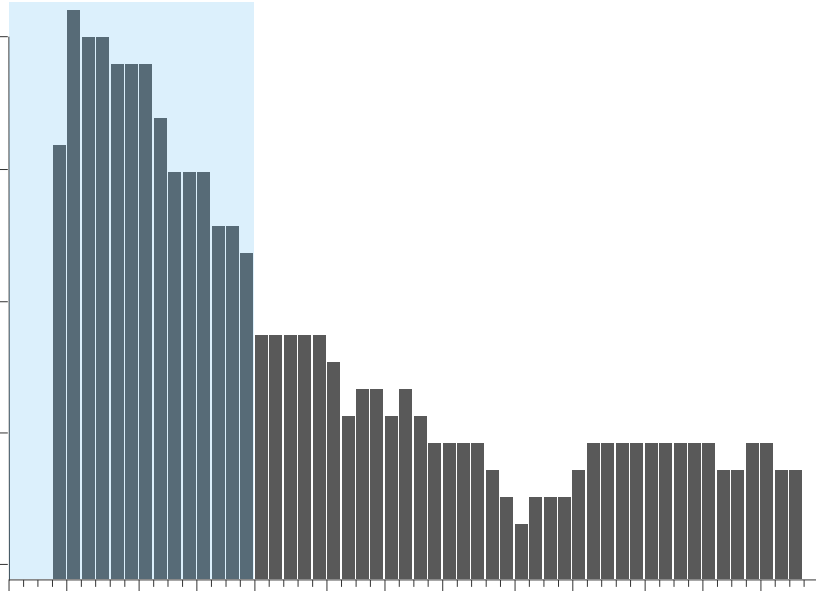

Supplement: Supplementary file 1 — Fig S1 [file ECE3-10-4716-s001.pdf]

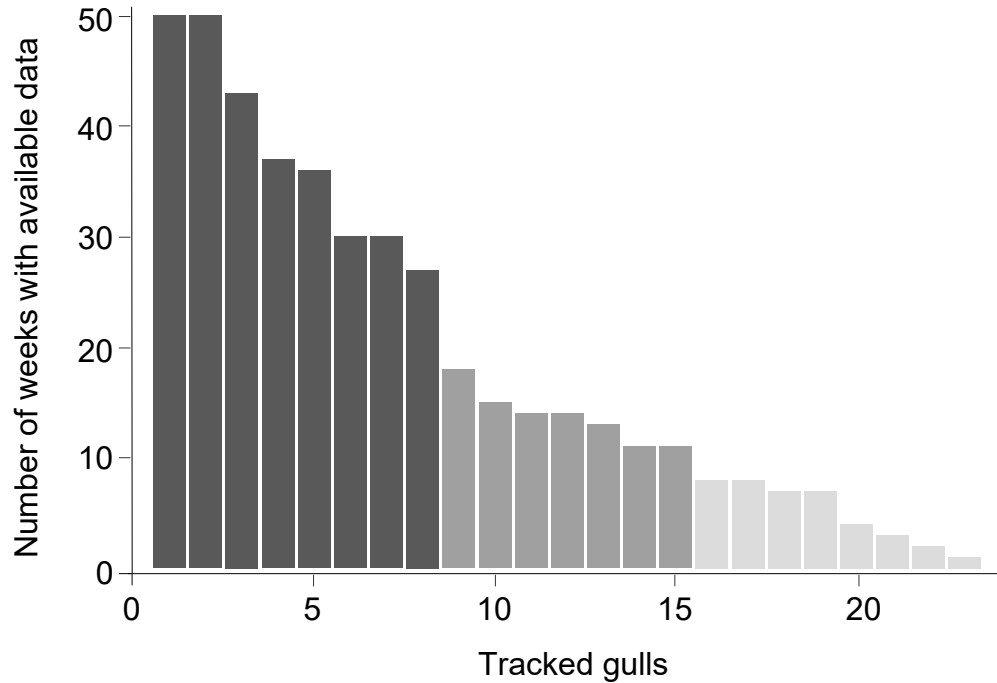

Supplement: Supplementary file 2 — Fig S2 [file ECE3-10-4716-s002.pdf]
