# Supplementary material for: Humans shape the year‐round distribution and habitat use of an opportunistic scavenger
Source: Ecol Evol. 2020 Apr 15;10(11):4716–25. doi: 10.1002/ece3.6226 (PMC7297764; doi:10.1002/ece3.6226)
Supplement: Supplementary file 3 — Fig S3 [file ECE3-10-4716-s003.pdf]

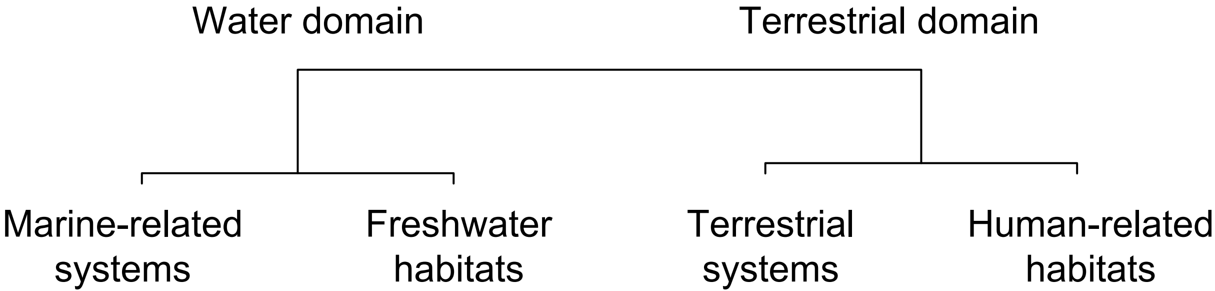
Water domain
Terrestrial domain
Marine-related systems
Freshwater habitats
Terrestrial systems
Human-related habitats
