# Supplementary material for: Humans shape the year‐round distribution and habitat use of an opportunistic scavenger
Source: Ecol Evol. 2020 Apr 15;10(11):4716–25. doi: 10.1002/ece3.6226 (PMC7297764; doi:10.1002/ece3.6226)
Supplement: Supplementary file 4 — Appendix S1‐S2 [file ECE3-10-4716-s004.docx]

**Appendix S1.** Analyses on missing patterns

During the incubation period of 2015 (April the 21^th^ to May the 7^th^), 30 adult gulls were equipped with solar-powered UvA-BiTS GPS trackers (<http://www.UvA-BiTS.nl>; Bouten et al. 2013). However, the number of tracked birds, and hence recorded data, declined throughout the annual cycle likely due to loss of devices, gull deaths or technical constraints during data retrieval (Appendix 1-Figure 1).


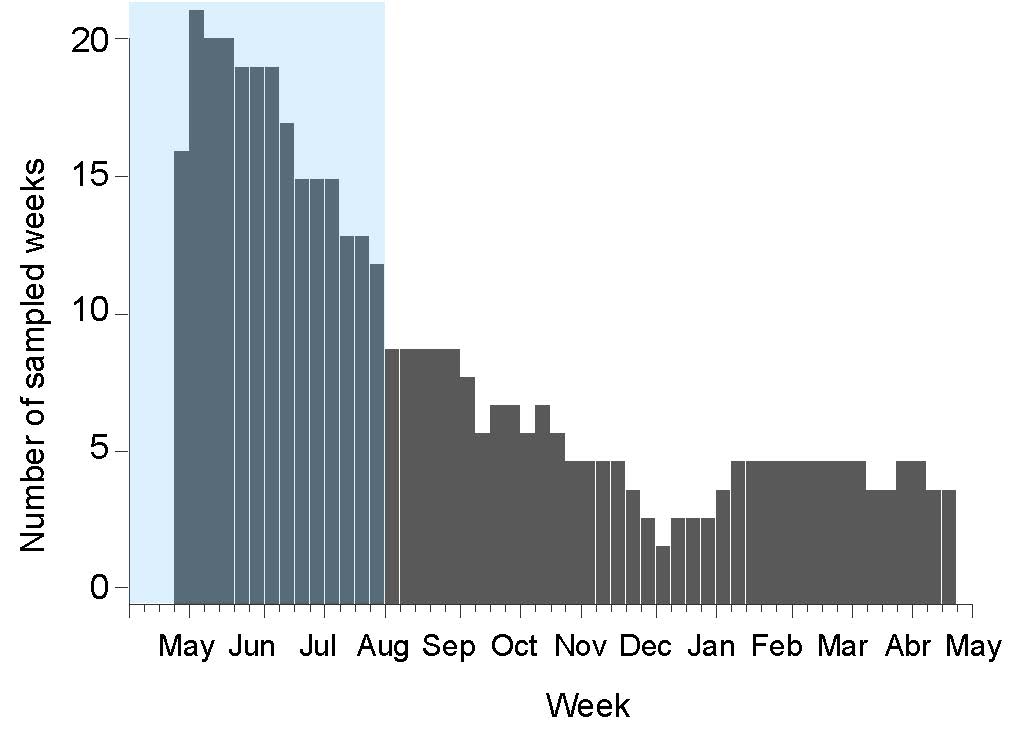


***Appendix 1-Figure S1.*** *Number of tracked gulls per week throughout the 2015-2016 annual cycle*

Our approach to evaluate the relative trends in habitat usage by tracked gulls assumes that missing information due to the decline in the number of tracked individuals throughout the study period was random and had no associations with the response variables we were fitting (i.e., habitat usage). In other words, we considered that observed trends in habitat associations were not biased by the better performance of individuals (and/or tracking devices) preferentially occurring at particular habitats. For testing this assumption, we evaluated patterns for missing data following Hedeker and Gibbons (2006). Individuals were first categorized in three different groups (missing group) depending on the number of weeks (our unit of time) with available information: 0-8 weeks (number of individuals = 8); 9-18 weeks (7); 19-52 weeks (8) (Appendix 1-Figure 2). We then used a Linear Mixed Models (LMMs) to evaluate differences in habitat usage for Isometric Log Ratio transformed relative proportions (Egozcue et al. 2003) among missing groups during the breeding period (April to July 2015; number if weeks with available data per missing group: 40, 86, 96, respectively). LMMs included the individual as a random factor and the missing group as a three-level fixed factor; which significance was tested through a Likelihood Ratio Test (LRT).


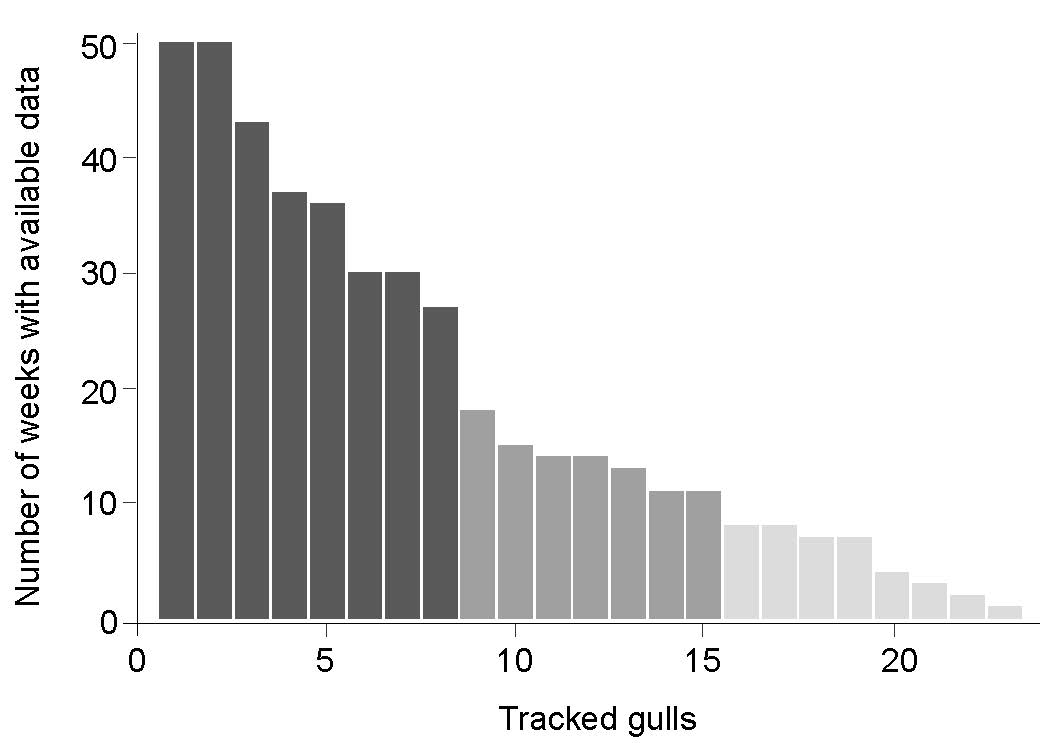


***Appendix 1-Figure S2.*** *Number of weeks with available data for tracked gulls**. Colours denote levels for the missing group factor.*

Overall, we did not find a significant effect of the missing group factor (Appendix 1-Table 1). This suggested that missing data for the entire annual cycle was random, thus pointing to the suitability of our approach to evaluate trends in habitat use based on maximum likelihood (Hedeker and Gibbons 2006).

|  |  |  | Model estiamtes | |
| --- | --- | --- | --- | --- |
| ILR proportions | *LRT*  *p*-value | Missing group | Mean | 95% CI |
| Water vs. Terrestrial domains | 0.98 | 0-8 | 2.21 | 1.4 - 2.85 |
|  |  | 9-18 | 2.03 | 1.28 - 2.77 |
|  |  | 19-52 | 2.05 | 1.36 - 2.74 |
| Freshwater habitats vs. Marine-related systems | 0.62 | 0-8 | -0.2 | -0.79 – 0.39 |
|  |  | 9-18 | -0.03 | -0.64 – 0.57 |
|  |  | 19-52 | 0.21 | -0.36 – 0.78 |
| Human-related habitats vs. Terrestrial systems | 0.65 | 0-8 | 0.83 |  |
|  |  | 9-18 | 0.41 | -0.41 – 1.24 |
|  |  | 19-52 | 0.92 | 0.15 – 1.69 |

***Appendix 1-Table S1.*** *Outputs for the Linear Mixed Models (LMMs) to evaluate differences in habitat usage for Isometric Log Ratio (ILR) transformed relative proportions among missing groups, a three-level fixed factor that classify tracked gulls depending on the number of weeks with available information: 0-8, 9-18 and 19-52 weeks (8). LMMs included the individual as a random factor and used the Likelihood Ratio Test (LRT) to evaluate the significance of the missing group factor.*

**Appendix S2.** Hierarchical clustering for habitat associations

We evaluated trends in habitat partitioning through compositional analyses based on Isometric Log Ratio (ILR) transformation of habitat proportions (in terms of time spent at each particular habitat category per week) (Egozcue et al. 2003). This approach requires pair-wise comparisons among habitat categories (freshwater habitats, marine-related systems, human-related habitats and terrestrial systems) to evaluate relative associations among habitat types. Pair-wise comparisons were based on a hierarchical clustering on habitat associations (Appendix 2-Figure 1). Similarities among habitats types were estimated through Centered Log-Ratios (clr) of habitat proportions (Aitchison 1986, Faith 2015). Clustering was performed by applying the *hclust* function from the *stats* R package (R Core Team 2017). Initially, each object was assigned to its own cluster and then the algorithm proceeded iteratively, at each stage joining the two most similar clusters, continuing until there was just a single cluster. At each stage, distances between clusters were recomputed by the Lance–Williams dissimilarity update formula according to the Ward's minimum variance method (Murtagh and Legendre 2014).


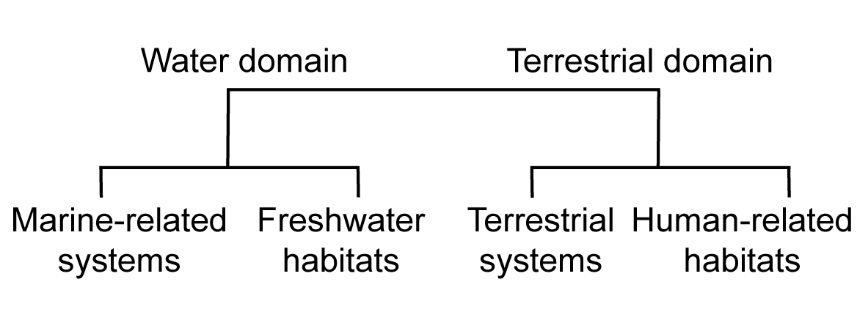


***Appendix 2-Figure S3.*** *Hierarchical cluster for habitat proportions based on Ward's minimum variance method (Murtagh and Legendre 2014). Similarities among habitats types were estimated through Centered Log-Ratios (clr) of habitat proportions*

Based on derived associations among habitat categories, trends in habitat use (i.e., weekly changes in relative habitat proportions) throughout the annual cycle were described modelled in three successive steps. We first evaluated the relative trends for the water (freshwater habitats and marine-related systems) *vs.* the terrestrial (human-related habitats and terrestrial systems) domains. Subsequently, we evaluated relative trends within each domain; i.e., freshwater habitats *vs.* marine-related systems and human-related habitats *vs.* terrestrial systems.
